# Investigation of the Interaction of Naringin Palmitate with Bovine Serum Albumin: Spectroscopic Analysis and Molecular Docking

**DOI:** 10.1371/journal.pone.0059106

**Published:** 2013-03-20

**Authors:** Xia Zhang, Lin Li, Zhenbo Xu, Zhili Liang, Jianyu Su, Jianrong Huang, Bing Li

**Affiliations:** 1 College of Light Industry and Food Sciences, South China University of Technology, Guangzhou, China; 2 Guangdong Province Key Laboratory For Green Processing Of Natural Products and Product Safety, South China University of Technology, Guangzhou, China; 3 Department of Microbial Pathogenesis, Dental School, University of Maryland, Baltimore, Maryland, United States of America; 4 School of Food Science, Guangdong Pharmaceutical University, Zhongshan, China; Aligarh Muslim University, India

## Abstract

**Background:**

Bovine serum albumin (BSA) contains high affinity binding sites for several endogenous and exogenous compounds and has been used to replace human serum albumin (HSA), as these two compounds share a similar structure. Naringin palmitate is a modified product of naringin that is produced by an acylation reaction with palmitic acid, which is considered to be an effective substance for enhancing naringin lipophilicity. In this study, the interaction of naringin palmitate with BSA was characterised by spectroscopic and molecular docking techniques.

**Methodology/Principal Findings:**

The goal of this study was to investigate the interactions between naringin palmitate and BSA under physiological conditions, and differences in naringin and naringin palmitate affinities for BSA were further compared and analysed. The formation of naringin palmitate-BSA was revealed by fluorescence quenching, and the Stern-Volmer quenching constant (*K_SV_*) was found to decrease with increasing temperature, suggesting that a static quenching mechanism was involved. The changes in enthalpy (Δ*H*) and entropy (Δ*S*) for the interaction were detected at −4.11±0.18 kJ·mol^−1^ and −76.59±0.32 J·mol^−1^·K^−1^, respectively, which indicated that the naringin palmitate-BSA interaction occurred mainly through van der Waals forces and hydrogen bond formation. The negative free energy change (Δ*G*) values of naringin palmitate at different temperatures suggested a spontaneous interaction. Circular dichroism studies revealed that the α-helical content of BSA decreased after interacting with naringin palmitate. Displacement studies suggested that naringin palmitate was partially bound to site I (subdomain IIA) of the BSA, which was also substantiated by the molecular docking studies.

**Conclusions/Significance:**

In conclusion, naringin palmitate was transported by BSA and was easily removed afterwards. As a consequence, an extension of naringin applications for use in food, cosmetic and medicinal preparations may be clinically and practically significant, especially in the design of new naringin palmitate-inspired drugs.

## Introduction

**Figure 1:**
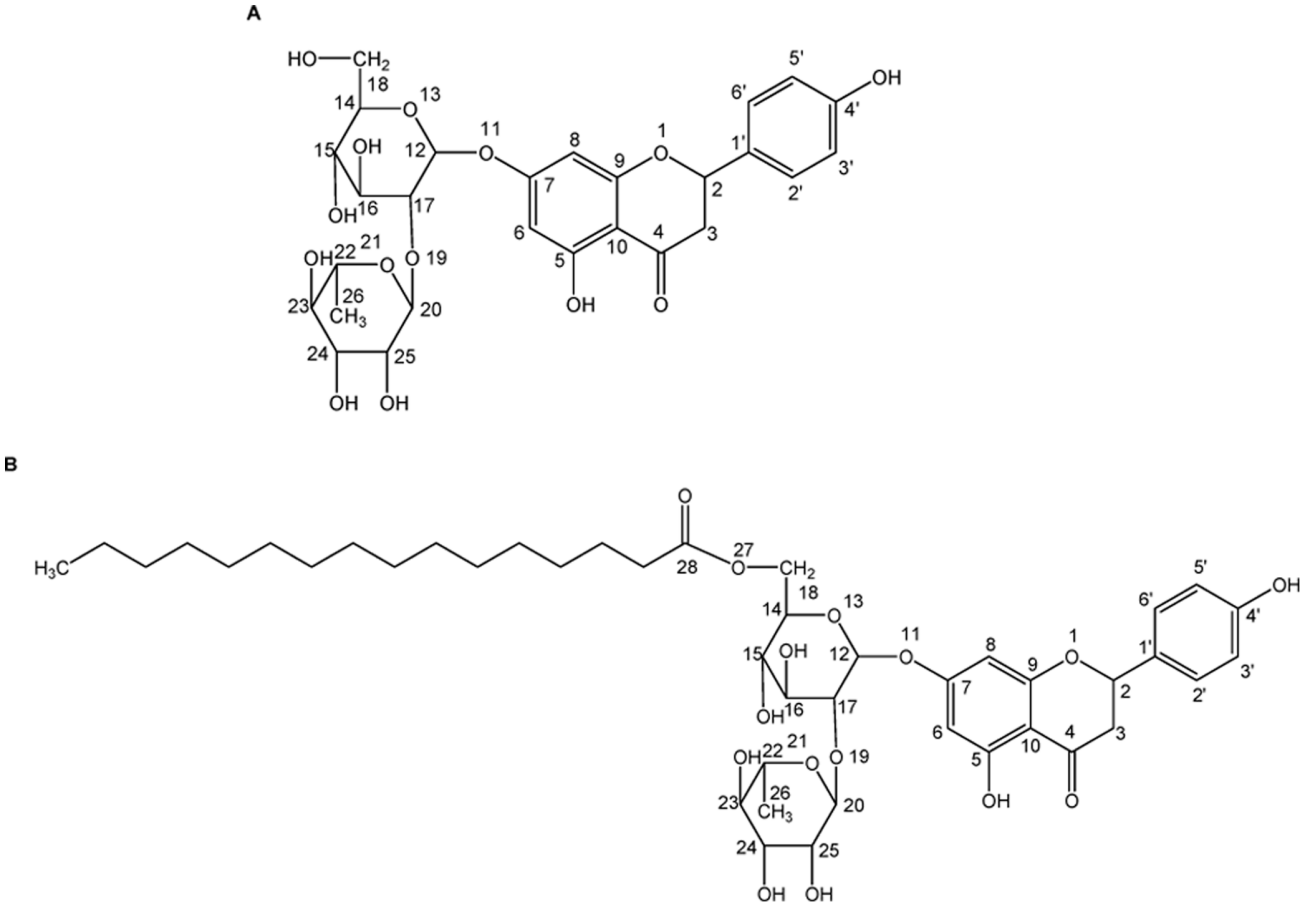
Chemical structure of naringin and naringin palmitate. (A) naringin and (B) naringin palmitate.

Naringin (4′, 5, 7-trihydroxy flavonone 7-rhamnoglucoside) is a flavonone glycoside (Fig. 1A) that is mainly found in grapefruit and other citrus fruits. It gives the grapefruit or citrus fruit typical bitter taste. As a member of flavonoid family, naringin exhibits effects such as antioxidant [1], anti-inflammatory [2], anti-microbial [3], hepatoprotective [4], and anticancer activities [5]. Naringin affinity for proteins, enzymes, DNA, RNA or particular cell types, as well as its ability to penetrate the cell membrane determines its biological effects [6]. In vitro experiments have been carried out to verify naringin binding to human serum albumin (HSA) and bovine serum albumin (BSA), as well as transport by these proteins, which is pivotal in the design of new naringin-inspired drugs [7]–[8]. However, the practical application of naringin has been limited by its poor solubility and stability in lipidic environments. Previous investigators tried to modify its degree of lipophilicity by inducing suitable chemical radicals into naringin structure [9]–[11]. Aliphatic groups were usually used to esterify flavonoid hydroxyl radicals in order to increase their solubility in oils, fats and lipophilic media, as well as their effectiveness. A naringin acylation reaction with a fatty acid is expected to effectively and manageably enhance its lipophilicity, thereby expanding its use in food, cosmetic and medicinal preparations. Palmitic acid is one of the most common saturated fatty acids in animals and plants and is widely used in esterification processes. Naringin palmitate has been synthesized by lipase-catalyzed acylation [11]–[12], and an ester bond from palmitic acid has been identified to present on the C-6′′ of the glucose moiety of the naringin glucose moiety (Fig. 1B). The degree of lipophilicity in naringin palmitate is higher than in naringin, a property that has exhibited an advantage in the transports and metabolic processes during drug therapy. However, the affinity and interaction mechanisms of naringin palmitate to proteins still remain poorly understood, which hinders its application in clinical treatment. Hence, further investigations of naringin palmitate are significant and will undoubtedly aid in understanding the transports and metabolic processes of modified-flavonones in herbal medicine.

Serum albumins are the major components of plasma proteins in all mammals [13] and serve as storage and transport proteins for many drugs and other bioactive small molecules [14]. Bovine serum albumin (BSA) has been studied extensively in kinetic and affinity drug tests as a replacement for human serum albumin (HSA) because of its easy accessibility, high stability, ability to bind various ligands and structural similarity to HSA [13]. Therefore, studying the effects of naringin palmitate on the solution structure of BSA will offer significant guidance concerning the interaction between naringin palmitate and protein, as well as drug development.

**Figure 2:**
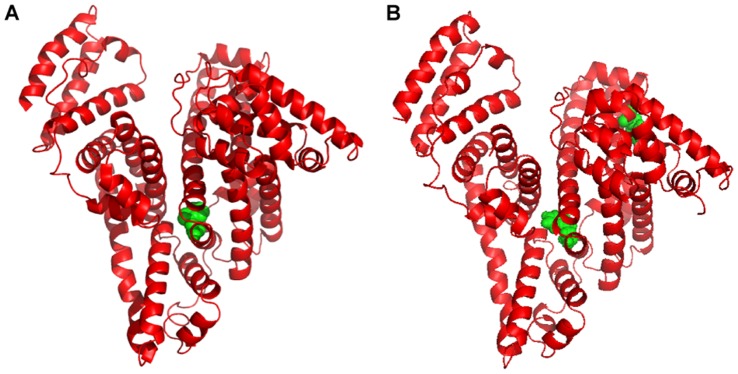
Three-dimensional structures of HSA and BSA with tryptophan residues in green color. (A) HSA (B) BSA.

BSA is composed of a single-chain 583 amino acid globular nonglycoprotrein that is cross-linked with 17 cystine residues (8 disulfide bonds and 1 free thiol) [15] and has been categorized into the following three homologous domains (I, II, III): I (residues 1–195), II (196–383), and III (384–583), which are divided into nine loops (L1–L9) by 17 disulfide bonds [16]. The loops in each domain consist of a sequence of large-small-large loops that form a triplet. There are two tryptophans in BSA, with Trp-134 located on the surface of subdomain IIA and Trp-213 embedded in the hydrophobic pocket of subdomain IB in a way that is similar to Trp-214 in HSA (Fig. 2) [17]. The molecular interactions between naringin, quercetin, rutin and other flavonoids with proteins have been extensively investigated [7], [8], [16], [18]–[22], and the interaction between structurally different flavonoids (including naringin) with HSA and BSA was previously investigated. Zhang et al. [7] studied the interaction mechanism between naringin and HSA and suggested that naringin could bind to Sudlow’s site I within subdomain IIA of HSA mainly by hydrophobic interactions. The interaction of naringin with BSA in a 20 mM phosphate buffer of a pH of 7.0 has been performed [8], and the naringin-BSA interaction was found to occur mainly through hydrophobic interactions, with a decrease in the helical content of BSA following its interaction with naringin and binding to site I (subdomain IIA) of BSA. The interaction results for the interaction of naringin with BSA and HSA are similar, with naringin binding to Sudlow’s site I within subdomain IIA of HSA or BSA, which occurs mainly through a hydrophobic interaction.

The induction of the palmitic acid chain by naringin would change the structure and solubility of naringin. The biological and pharmacological functions of naringin palmitate may be different from naringin. In this study, naringin palmitate was been synthesized in organic solvent. It was studied using fluorescence spectroscopy, UV absorption spectroscopy, FT-TR, CD spectroscopy, site maker displacement and molecular modeling methods. Investigations of the binding constant (*K*), the number of binding sites (*n*), thermodynamic parameters for the reaction, and changes to the binding sites and conformation in BSA in response to naringin palmitate were performed. The fact that these are the first spectroscopic results for naringin palmitate-BSA interactions illustrates the complex nature of this subject. A comparison between naringin palmitate-BSA and naringin-BSA was performed in order to confirm naringin palmitate functionality.

## Materials and Methods

### Materials

Immobilized lipase Novozyme 435 (immobilized lipase from *Candida antarctica*) was purchased from Sigma (Germany). Naringin was sourced from Fluka (ref. 71162, Switzerland). Palmitic acid (Fluka, Switzerland) was used as an acyl donor. The *tert*-amyl alcohol (Merck) was used as solvents during esterification. Molecular sieves of 4 Å (8–12 mesh beads), warfarin and ibuprofen were purchased from Sigma (United States of America). Bovine serum albumin was purchased from Sino-American Biotechnology Company (Beijing, China) and used without further purification. The stock solution of naringin and naringin palmitate (5×10^−3^ M) was prepared in dimethyl sulfoxide (DMSO). Stock solutions of warfarin(1.5×10^−3^ M) and ibuprofen (1.0×10^−3^ M) were prepared [23]. A BSA stock solution (1.50×10^−5^ M) was prepared in 0.1 M NaCl solution. The buffer solution (pH 7.40) was composed of Tris-HCl (0.1 M). All stock solutions were kept in the dark at 4°C and then diluted to the required experimental sample concentrations. All BSA samples were prepared in the pH 7.40 buffer solution. The concentrations of BSA, warfarin and ibuprofen in the spectroscopic samples were determined by UV absorbance measurements using an extinction coefficient of 43800 M^−1 ^cm^−1^ at 280 nm for BSA [24]–[25], 12500 M^−1^cm^−1^ at 308 nm for warfarin [26], and 172700 M^−1 ^cm^−1^ at 308 nm for ibuprofen [27]. The concentrations of naringin and naringin palmitate were determined by high performance liquid chromatography (HPLC). All other reagents were of analytical grade, and double distilled water was used in all experiments.

#### Enzymatic synthesis and purification of naringin palmitic acid esters

The enzymatic synthesis of naringin palmitate in organic solvents was carried out in a sealed vial of 50 mL in order to prevent solvent evaporation. The vial was incubated in an orbital shaker at 150 rpm at 60°C. In a typical acylation reaction, naringin (150 mM) and palmitic acid (250 mM) were mixed in 5 mL of solvents with addition of 4 Å molecular sieves (100 g/L) which were activated by heating at 180°C for 1.5 h prior to use. Naringin acylation was started with the addition of immobilized lipase. Parallel experiments were carried out at a predetermined time interval, and one vial was withdrawn to determine the concentrations of substrate and products by high performance liquid chromatography (HPLC). The reaction was terminated by adding 5 mL of methanol and removing the immobilized enzyme and molecular sieves by filtration. After the reaction was terminated, the naringin palmitic acid esters were purified by column chromatography (100–200 mesh, 10×600 mm) (Zhenghui Company, Shanghai, China) on silica gel 60 A, using a mixture of ethylacetate/methanol/methanoic acid (15/1/1, v/v/v) as eluents.

#### Fluorescence Quenching Studies

All fluorometric experiments were recorded on an F-2500 spectrofluorometer (HITACHI, Japan) using a 1 cm quartz cell and a thermostat bath. Ten millilitres of 1.5 µM BSA solution was prepared and titrated with increasing concentrations of naringin and naringin palmitate (0 to 50 µM) in 5 mM buffer solution at three different temperatures (288, 298 and 310 K). An appropriate blank that corresponded to the buffer was subtracted in order to correct for background fluorescence. The excitation wavelength was 282 nm, and the emission spectra were recorded from 300 to 450 nm. The widths of both the excitation and the emission slit were set to 5.0 nm. The maximum emission intensities were used to calculate binding constants, occupation of binding sites and thermodynamic parameters.

Fluorescence quenching is the decrease of quantum yield of fluorescence from a fluorophore that is induced by a variety of molecular interactions with a quencher molecule [28]. The quenching process is described by the Stern-Volmer equation [29].
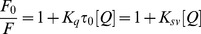
(1)Where *F_0_* and *F* are the relative fluorescence intensities in absence and presence of the quencher, *τ_0_* is the lifetime of the fluorophore in absence of quencher with a value of 10^−8^ s [30], and *k_q_* is the quenching constant of bimolecular. *K_SV_* is the Stern-Volmer quenching constant and *[Q]* is the quencher concentration. *K_SV_* was obtained by plotting *F_0_/F* versus *[Q]*, and if *K_SV_* is much larger than 2×10^10^ M^−1 ^s^−1^, then the quenching is static in nature [8].

Small molecules are assumed to bind independently to a set of equivalent sites on a macromolecule, and the number of binding sites and the binding constant of the interaction between naringin and BSA were found with the following equation [31]:
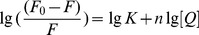
(2)where *K* is the binding constant for a site and *n* is the number of bindings per albumin, respectively. The plot of *lg ((F_0_–F)/F_0_)* versus *lg [Q]* yields *lg K* as the intercept and *n* as the slope. The values for *K* and *n* can be obtained from the intercept and slope.

Interaction forces between quencher and biomolecules may include hydrophobic force, electrostatic interactions, van der Waals interactions, hydrogen bonds, and others [15]. In order to map the interaction of naringin/naringin palmitate with BSA, the thermodynamic parameters were calculated using the Van’t Hoff equation [32]–[33]:
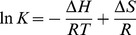
(3)where *K* is the binding constant to a site, *R* is the universal gas constant (8.314 J·mol^−1^·K^−1^), Δ*H* and Δ*S* are the changes in enthalpy and entropy during quenching process.

The free energy change (Δ*G*) associated with the interaction of naringin/naringin palmitatewith BSA can be calculated from the following equation:
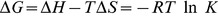
(4)


The energy transfer between naringin/naringin palmitate and BSA can be calculated according to Föster’s non-radiative energy transfer theory [34] when an acceptor absorbs the fluorescence that was emitted from a donor. The distances between donor (BSA) and acceptor (naringin/naringin palmitate) can therefore be determined. The efficiency of energy transfer as it relates to this distance from the following equation [35]:
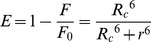
(5)where *E* represents the energy transfer efficiency between the donor and the acceptor, *r* is the distance between the donor and acceptor, and *R_c_* is the critical distance when the efficiency of energy transfer is 50%. The value of *R_c_^6^* can be calculated using the following equation [34]:
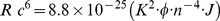
(6)where K2 is the spatial orientation factor related to the geometry of the donor and acceptor of dipoles, and K2 is 2/3 for random orientation as in fluid solution; φ is the fluorescence quantum yield of the donor, n is the averaged refracted index of the medium (usually resulting in a value of 1.336, which is the average for water and organic matter), and *J* is the spectral overlap integral between the fluorescence emission spectrum of the donor and the absorption spectrum of the acceptor. The value of *J* can be calculated using the following equation [34]:
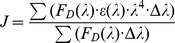
(7)where FD(λ) is the corrected fluorescence intensity of the donor in the wavelength range from λ to λ+Δλ, ε(λ) is the molar absorption coefficient of the acceptor at wavelength λ, and Δλ is the span of the wavelength.

### Synchronous Fluorescence Quenching

Synchronous fluorescence spectra of BSA (1.50×10^−6^ Μ) were recorded with increasing concentrations of naringin/naringin palmitate (0 to 50 μΜ), by setting Δλ = 60 nm and Δλ = 15 nm for tryptophan and tyrosine residues respectively [36]. The widths of both the excitation and the emission slit were set to 5.0 nm.

### Site Marker Competitive Experiments with Warfarin and Ibuprofen

For the competitive warfarin/ibuprofen BSA experiment, 10 mL of solution was prepared with warfarin (1.5 µM) or ibuprofen (1.0 µM) and BSA and then allowed to stand for 1 hour at 288 K [8]. Titrations were performed with increasing concentrations of naringin and naringin palmitate (0 to 50 µM). The excitation wavelengths of warfarin-BSA and ibuprofen-BSA were 308 nm and 265 nm [8]. The fluorescence quenching data were analyzed using the Stern-Volmer equation [37].

### UV/vis Absorption Spectra

The absorption spectra were measured on a U-3010 spectrophotometer (HITACHI, Japan) with a 1.0 cm path length cell. The UV/vis absorption spectra were obtained by scanning the solution containing 1.5 µM BSA solution while increasing the concentrations of naringin and naringin palmitate (0 to 50 µM) in the spectrophotometer with the wavelength range of 295–495 nm. Analyses were carried out at room temperature.

### Circular Dichroism (CD) Measurements

All BSA far-UV CD spectra with different amounts of naringin/naringin palmitate were obtained with a MOS-450 spectropolarimeter (Bio-Logic Science Instruments, Grenoble, France) in cylindrical cells (2 mm path length) at 288 K. The concentration of BSA in the CD study was 1.5 µM in 5 mM Tris-HCl buffer with a pH of 7.4. Seven sets of solutions were prepared with BSA and naringin/naringin palmitate in the following proportions 1∶0, 1∶2, 1∶4, 1∶6, 1∶8, 1∶10 and 1∶15. The spectra were recorded from 190 to 250 nm with step resolutions of 0.5 nm, an acquisition duration of 1 s, bandwidth of 5.0 nm and sensitivity of 100 mdeg during all measurements. The spectra in the far-UV (190–250 nm) region required an average of six scans. The spectrum of the buffer and naringin/naringin palmitate control solution was subtracted from the sample spectra. The CD measurements were expressed in terms of mean residue ellipticity (*MRE*) in deg cm^2^ dmol^−1^, which can be estimated with Eq. 8 [38]–[39].
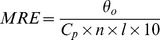
(8)Where *θ_o_* is the observed CD value, *C_p_* is the molar concentration of the protein (1.5 µM), *n* is the number of amino acid residues in the protein (583) and *l* is the path length of the cell (2 mm). The α-helical contents of free and complexed BSA were calculated from the mean residue ellipticity values at 208 nm using the following equation and as described previously [38]:
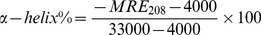
(9)where MRE208 is the observed MRE value at 208 nm, 33000 is the MRE value of a pure α-helix, and 4000 is the MRE value of the β-form and random coil conformation at 208 nm respectively.

### FT-IR Spectroscopic Measurements

Fourier-transfer infrared spectroscopy (FT-IR) was carried out on a Tensor 37 spectrometer (Bruker Optik, Madrid, Spain) that was equipped with a DTGS detector, a KBr beam splitter, and a Ge attenuated total reflection (ATR) accessory at room temperature. A 128-scan interferogram with a 4 cm^−1^ resolution was used to collect the spectra. The concentration of BSA for this experiment was 0.15 mM in 5 mM Tris-HCl buffer at a pH of 7.4. Three additional sets of samples were prepared with molar ratios of BSA to naringin/naringin palmitate of 1∶1, 1∶2 and 1∶3. The background was corrected before scanning, and the blank spectrum with the same buffer and naringin/naringin palmitate was prepared. The blank spectrum was subtracted from the BSA-naringin/naringin palmitate combined spectra. Infrared spectra were obtained in the 1800−1400 cm^−1^ featured region [40].

### Molecular Modeling and Docking

The crystal structure of BSA was obtained from the Protein Data Bank (entry code 4F5S) [41]. The PyMol molecular graphics system (DeLano Scientific, San Carlos, USA, Version 0.99) was used to delete water molecules from the crystal structure of BSA and visualise the docked conformation. The coordinate files of naringin/naringin palmitate were prepared and energy was minimized using the PRODRG server (http://davapc1.bioch.dundee.ac.uk/prodrg/index.html). Molecular docking simulations of naringin and naringin palmitate were performed with the AutoDock 4.2 program. Each grid computation was performed with a grid box of 60×60×60 Å with 0.375 Å spacing, which covered all the active site residues and allowed for the flexible rotation of the ligand. For rigid docking simulations, the parameters were set to 100 GA runs that terminated after a maximum of 2,500,000 energy evaluations, and the population size was set to 150 with a crossover rate of 0.8 (Lamarkian Genetic Algorithm). For flexible docking, the above parameters were the same, while some important neighbouring residues at the binding sites were set to flexible. The lowest energy conformation in the largest cluster of each docking simulation was extracted and analysed. The hydrogen bonds and hydrophobic interactions between the ligands and the protein were represented with Ligplot version 4.5.3 [42].

## Results and Discussion

### The Mechanism of BSA Fluorescence Quenching by Naringin and Naringin Palmitate

**Figure 3:**
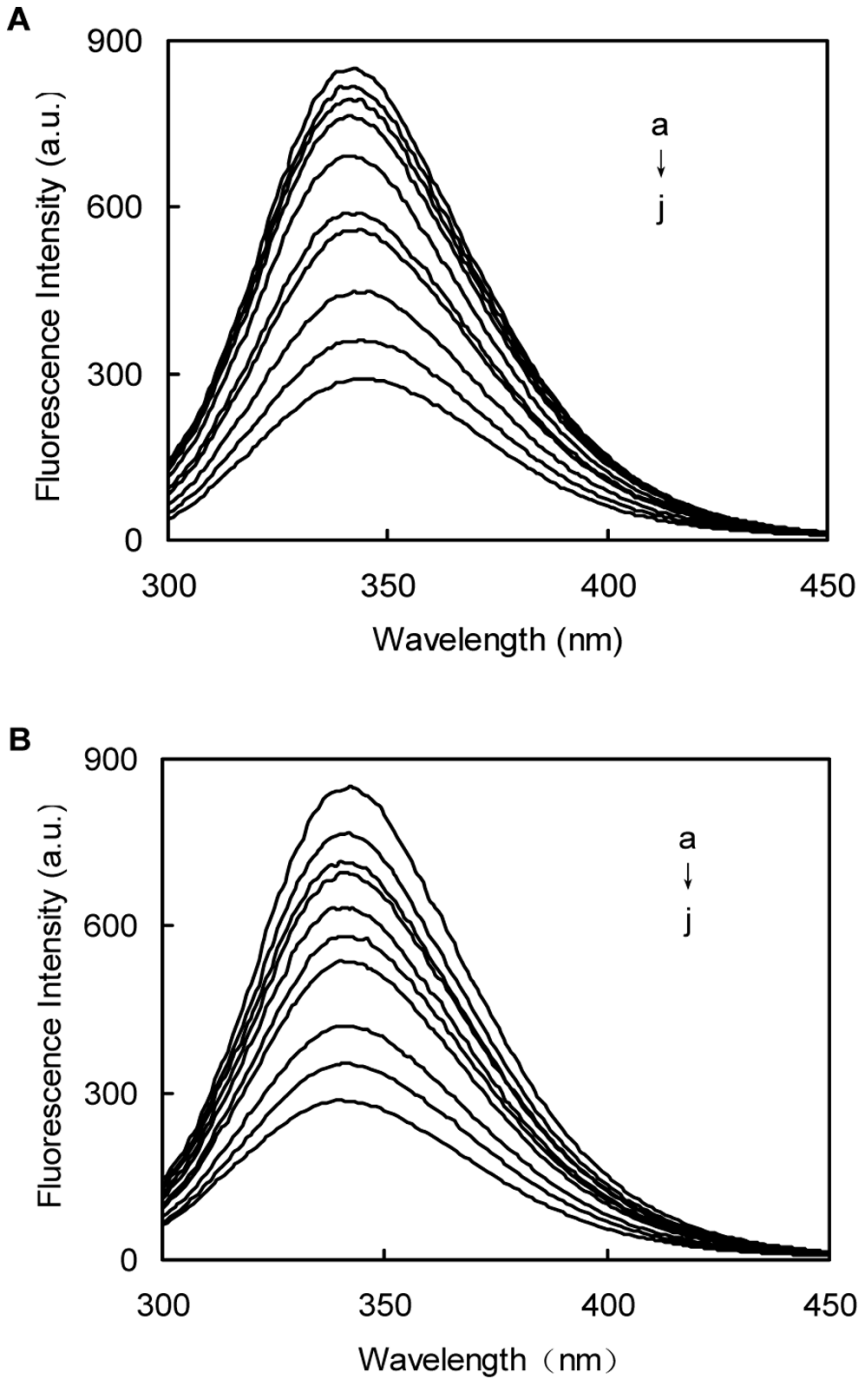
Fluorescence emission spectra of BSA-naringin and BSA-naringin palmitate in phosphate buffer pH 7.4. (A) 0, 1.5, 2.5, 5.0, 10, 15, 20, 30, 40, 50×10^−6^ M (curves a→j) of naringin treated with BSA (1.50×10^−6^ M) and (B) 0, 1.5, 2.5, 5.0, 10, 15, 20, 30, 40, 50×10^−6^ M (curves a→j) of naringin palmitate treated with BSA (1.50×10^−6^ M). λ_ex_ = 282 nm, temperature = 15±1°C.

The increasing concentration effects of naringin and naringin palmitate (0 to 50 μΜ) on BSA fluorescence intensity at 288 K is shown in Fig. 3. This figure demonstrates that BSA had a strong fluorescence emission peak at 342 nm after being excited with a wavelength of 282 nm with naringin palmitate and naringin under the experimental conditions, while naringin palmitate and naringin had no intrinsic fluorescence at the 282 nm excitation wavelength. When a fixed concentration of BSA was titrated with increasing amounts of naringin palmitate and naringin, a remarkable intrinsic fluorescence decrease of BSA was observed (Fig. 3). This finding indicated that naringin palmitate and naringin binding with BSA caused microenvironment changes in BSA and produced BSA-naringin and BSA-naringin palmitate complexes. Similar fluorescence quenching results were reported for several other ligands [43]–[44]. For naringin palmitate and naringin, the BSA fluorescence intensity of naringin palmitate was higher than in naringin, indicating that the fluorescence quenching of BSA by naringin was stronger than in naringin palmitate. The difference between the BSA fluorescence intensity of naringin palmitate and that of naringin decreased when the quencher concentration was increased. Some differences were observed between naringin and naringin palmitate during BSA binding, which may be related to the larger volume of naringin palmitate.

**Figure 4:**
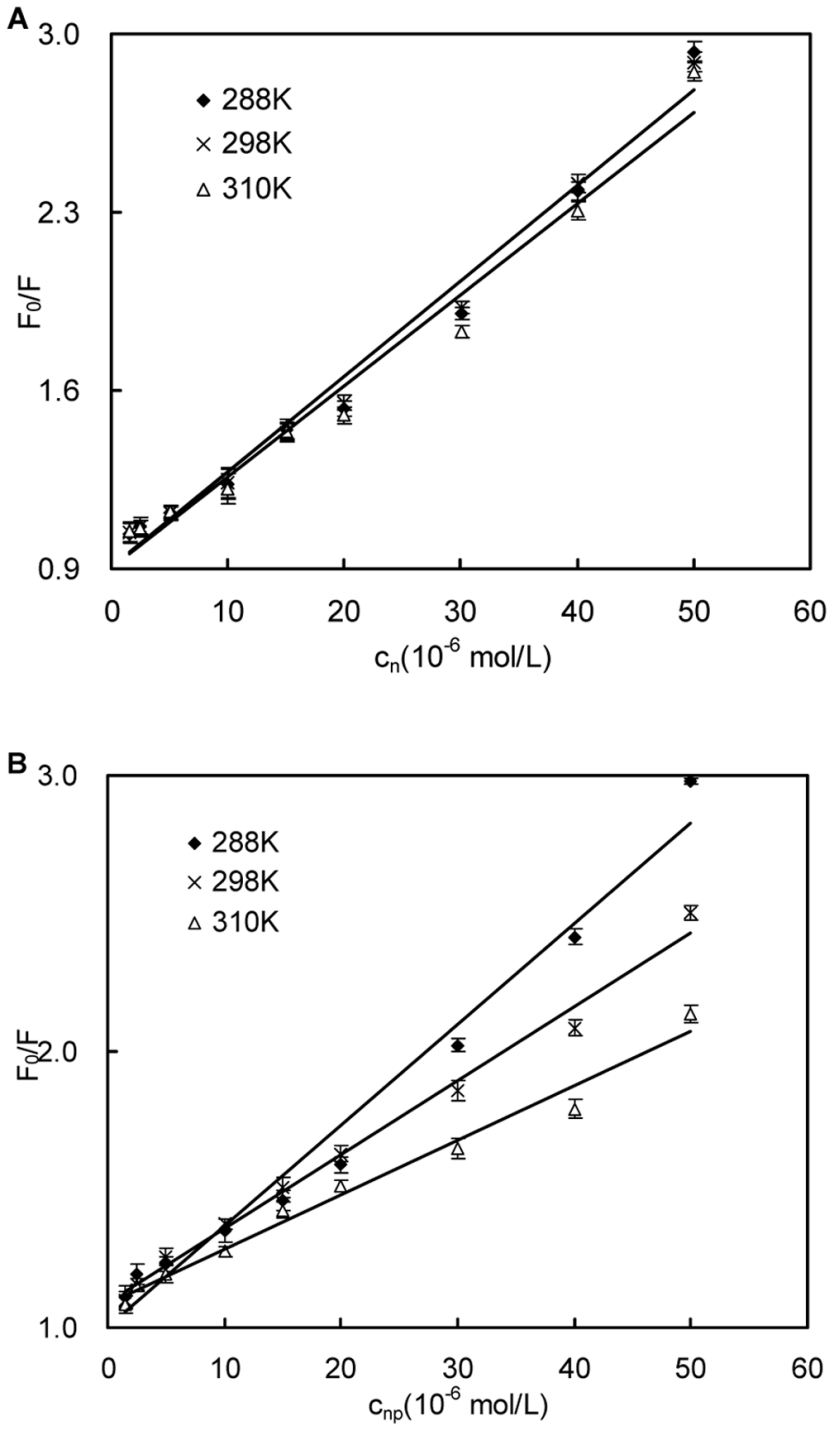
Stern-Volmer plots for BSA-naringin and BSA-naringin palmitate. (A) naringin and (B) naringin palmitate.

To illustrate the differences in BSA fluorescence quenching mechanism between naringin and naringin palmitate, the fluorescence spectra data were analyzed using the Stern-Volmer equation (Eq. 1) [45]. The interaction between naringin/naringin palmitate and BSA at different temperatures (288 K, 298 K and 310 K) was also studied (Fig. 4), and a strong linear relationship between *F*_0_/*F* and the concentration *c*_n_ was obtained. The Stern-Volmer equation values for naringin and naringin palmitate at different temperatures (288 K, 298 K and 310 K) are shown in Table 1.

**Table 1:** Stern-Volmer equation for the interaction between naringin and BSA.

Sample	T/K	*10^4^×K*_sv_/(L·mol^−1^)	*10^12^×K*_q_/(L·mol^−1^·s^−1^)	*R* a
Naringin	288	3.762±0.022	3.762±0.022	0.980
	298	3.744±0.031	3.744±0.031	0.986
	310	3.572±0.102	3.572±0.102	0.975
Naringinpalmitate	288	3.650±0.061	3.650±0.061	0.978
	298	2.683±0.017	2.683±0.017	0.991
	310	1.974±0.072	1.974±0.072	0.983

a R is correlation coefficient.

Table 1 also shows the Stern-Volmer static quenching constants (*K_SV_*) for naringin and naringin palmitate, which were inversely correlated with temperatures. Generally, dynamic quenching and static quenching exist within fluorescence quenching according to the difference of fluorescence quenching way [44]. To distinguish between dynamic and static types, the temperature effects on the interaction between the drug (naringin palmitate and naringin) and BSA were further examined. The Stern-Volmer dynamic quenching constant *K_SV_* increased with increasing temperature, while the static quenching constant *K_SV_* decreased with increasing temperature [46]. Therefore, the probable fluorescence quenching mechanism of BSA by naringin and naringin palmitate was static quenching. In addition, instead of dynamic collision, the fluorescence quenching of BSA by naringin and naringin palmitate resulted from compound formation, which was supported by the evidence that dynamic quenching was caused by a collision between quenching agents, while the static quenching was caused by compound generation [46]–[47].

On the other hand, the maximum collision quenching constant *K_q_* of different quencher with biopolymer was 2×10^10^ L·mol^−1^·s^−1^ [48], and the rate constants for the quenching of BSA by naringin and naringin palmitate increased (Table 1), confirming that the fluorescence quenching of BSA by naringin palmitate and naringin was a consequence of static quenching. These results were consistent with a previous study, in which Roy et al. studied the interaction of the antioxidant naringin with BSA [8]. Other flavonoids, hesperidin, rutin and quercetin had binding affinities of 5.445×10^4^ L·mol^−1^ (288 K) [18], 1.07×10^4^ L·mol^−1^ and 1.34×10^5^ L·mol^−1^ (298 K) [49].

For naringin and naringin palmitate, the Stern-Volmer static quenching constant *K_SV_* of naringin palmitate was lower than in naringin (Table 1), suggesting that naringin palmitate-BSA binding was weaker than naringin alone because higher *K_SV_* represented the stronger drug and BSA binding [48], which could mean that the large volume of naringin palmitate may restrict the entrance to the BSA interior. The Stern-Volmer static quenching constant *K_SV_* of naringin and naringin palmitate decreased with increasing temperature (Table 1), and the *K_SV_* of naringin palmitate was significantly affected by temperature, which was likely to lower the stability of the naringin-BSA and naringin palmitate-BSA system. The naringin palmitate-BSA complex was more sensitive to high temperature than the naringin-BSA complex. When used in the mammal bodies, naringin palmitate will be released from serum albumin more quickly than naringin, and will arrive more quickly at the maximum concentration. This finding may be used to improve short term clinical treatments.

### Binding Sites and Binding Constant

**Figure 5:**
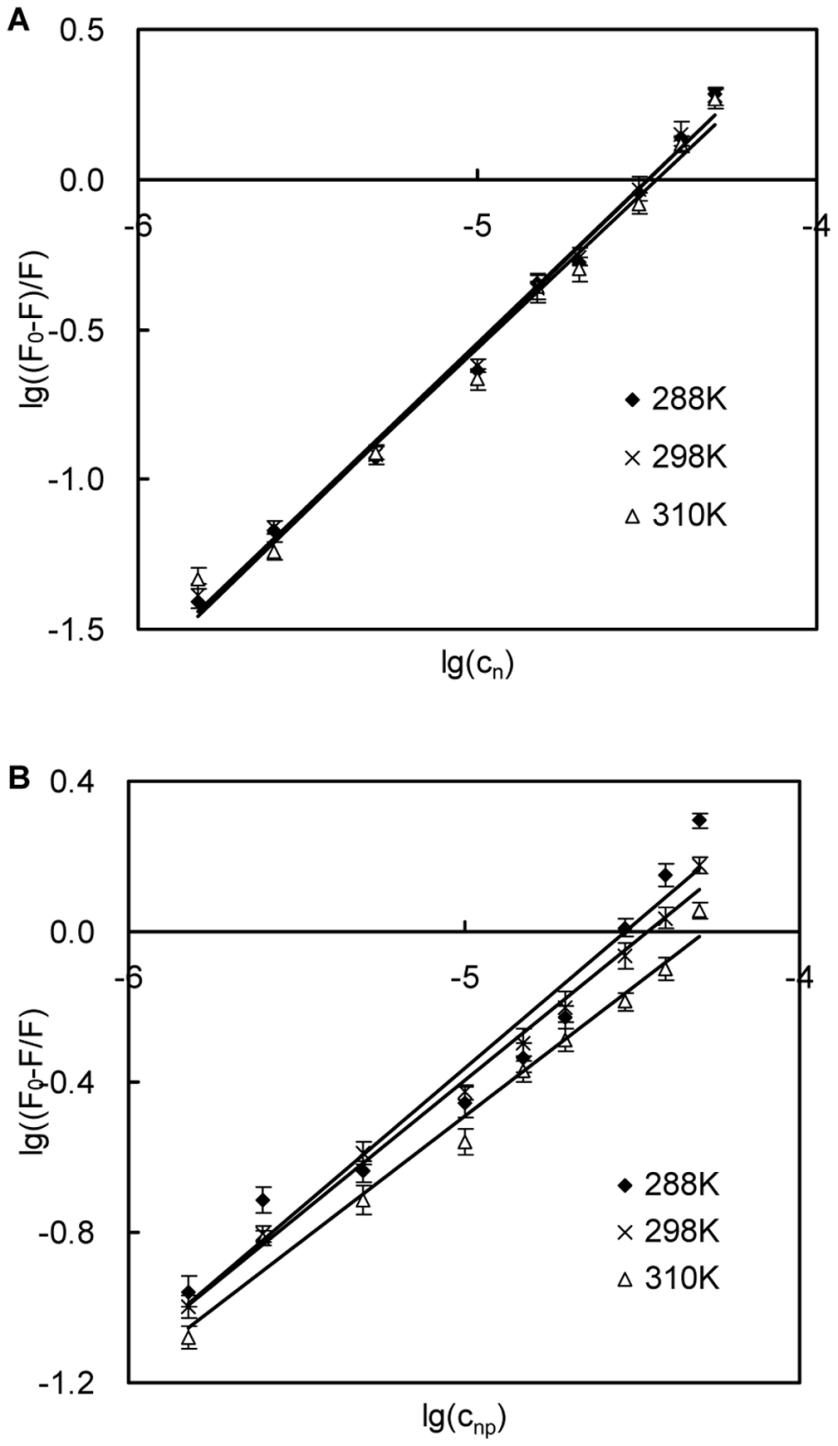
Scatchard plots for the interaction between naringin/naringin palmitate and BSA. (A) naringin and (B) naringin palmitate.

Fig. 5 shows the plots of *lg(F_0_–F)/F* vs. the concentration of naringin *lgc_n_* or the concentration of naringin palmitate *lgc_np_* at different temperatures (288 K, 298 K and 310 K). The binding constants *K* and binding sites *n* for naringin and naringin palmitate with BSA were calculated using Eq. 2, and the results are listed in Table 2. The values of *n* that were approximately equal to 1 corresponded to the presence of a single binding site in BSA [16], [18], and the ligands were most likely bound to Sudlow’s site I within the hydrophobic pocket of subdomain IIA [31], suggesting that Trp-213 was near or within the binding site [50]. The number of binding sites for naringin palmitate was lower than 1, meaning that naringin palmitate was partially bound to BSA and most likely bound to site I [27] (Table 2). The binding constant of naringin palmitate decreased with increasing temperature, indicating that naringin palmitate-BSA was not moderate and sensitive to temperature. In the case of naringin-BSA, the number of binding sites was higher than 1 and stable with increasing temperature, which corresponded to the high affinity sites that were involved in creating a strong binding force between the naringin and the BSA [51]. This finding indicated that naringin was most likely bound to site I in subdomain IIA with stable binding, and the temperature had a small effect despite the constant decrease in binding as the temperature increased. This result was also supported by the binding of naringin to HSA [7] and BSA, where it was also found to bind to the same site [8]. The number of binding sites and binding constant for naringin palmitate-BSA were less than in naringin-BSA, suggesting that the binding ability of naringin palmitate to BSA was weaker than that of naringin and that the distance from naringin palmitate to the binding site was farther than in naringin.

**Table 2:** Binding sites number (*n*) between BSA and naringin or naringin palmitate.

Sample	T/K	*10^4^×K*/L•mol^−1^	*n*	*R* a
Naringin	288	8.433±0.131	1.1	0.991
	298	7.727±0.182	1.1	0.992
	310	5.649±0.225	1.1	0.986
Naringin palmitate	288	0.279±0.015	0.8	0.955
	298	0.171±0.003	0.7	0.993
	310	0.083±0.002	0.7	0.983

a R is correlation coefficient.

### Binding Mode

**Figure 6:**
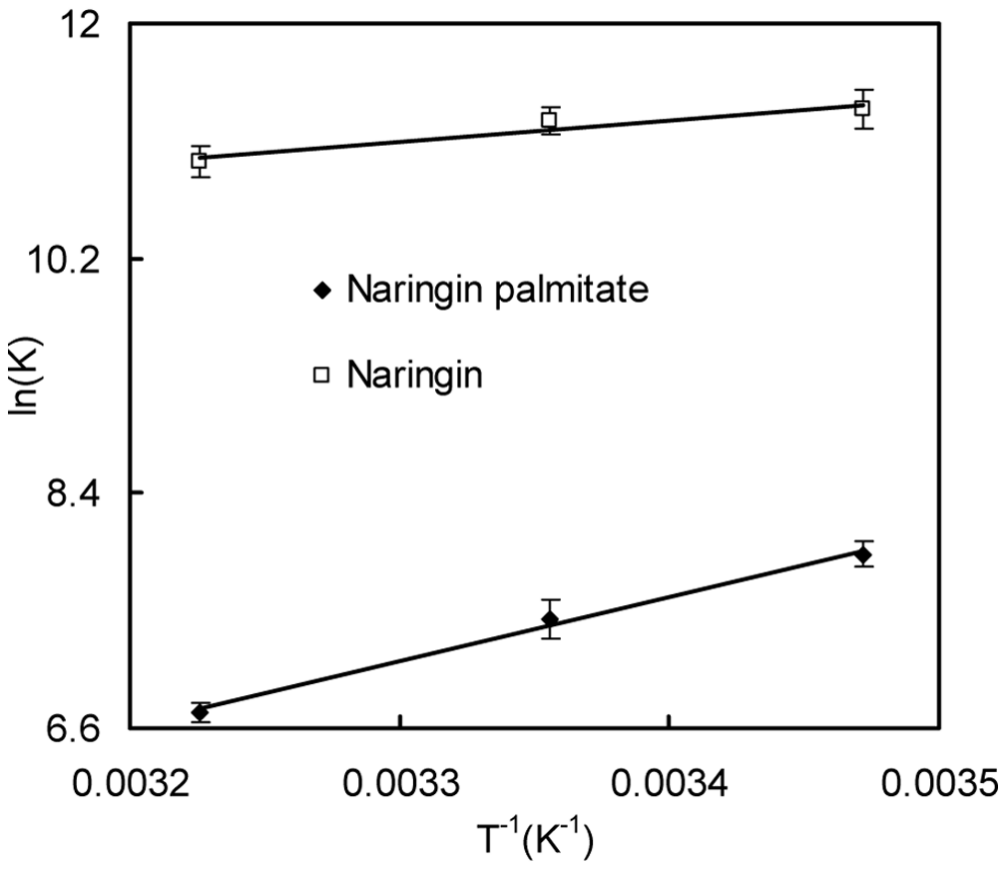
Van′t Hoff plots for the interaction between naringin/naringin palmitate and BSA. (A) naringin and (B) naringin palmitate.

According to the binding constants that were found at three temperatures above (288 K, 298 K and 310 K), the change in enthalpy (Δ*H*) and entropy (Δ*S*) values were obtained from linear Van’t Hoff plots (Fig. 6) with Eq.3, and the results are presented in Table 3. The free energy change Δ*G* was calculated with the Eq. (4). As Table 3 shown, the Δ*H* and Δ*S* for the binding reaction between naringin palmitate and BSA were found to be −4.11±0.18 kJ·mol^−1^ and −76.59±0.22 J·mol^−1^·K^−1^, and the Δ*H* and Δ*S* for the binding reaction between naringin and BSA were found to be −13.64±0.46 kJ·mol^−1^ and 47.23±0.67 J·mol^−1^·K^−1^. The negative Δ*G* corresponded to a spontaneous binding process [52]. Therefore, the binding process between naringin palmitate/naringin and BSA was spontaneous.

**Table 3:** Thermodynamic parameters of the interaction between BSA and naringin or naringin palmitate.

Sample	T/K	Δ*H*/(kJ·mol^−1^)	Δ*S*/(J·mol^−1^·K^−1^)	Δ*G*/(kJ·mol^−1^)	*R* a
Naringin	288	−13.64±0.46	47.23±0.67	−27.24±0.49	0.922
	298			−27.72±0.33	
	310			−28.28±0.21	
Naringin palmitate	288	−4.11±0.18	−76.59±0.22	−19.06±0.35	0.993
	298			−18.30±0.42	
	310			−17.38±0.14	

a R is correlation coefficient.

The interaction forces between quencher and biomolecules may include hydrophobic force, electrostatic interactions, van der Waals interactions, hydrogen bonds, and others [15]. A different sign and magnitude for the thermodynamic parameters appeared with different interaction forces [32]–[33], such as typical hydrophobic interactions of both positive Δ*H* and Δ*S*, van der Waals force and hydrogen bonding formation showing with negative Δ*H* and Δ*S* in low dielectric media, and electrostatic interactions playing a role in negative Δ*H* [53]. Therefore, the interaction forces behind the binding of naringin palmitate with BSA may derive from van der Waals force and hydrogen bonding formation because of the negative Δ*H* and Δ*S* (Table 3) [24]. The binding process of naringin-BSA involved electrostatic interaction as evidenced by the negative values of Δ*H* (Table 3). However, the change in entropy (Δ*S)*, rather than the change in enthalpy (Δ*H)*, provided a major contribution to Δ*G* [8], so the main forces in naringin-BSA binding were hydrophobic interactions because of the positive Δ*S*. This interaction was more likely hydrophobic, with electrostatic interactions involved in the naringin-BSA binding process [18]. In addition, the main interaction forces between naringin-BSA and naringin palmitate-BSA were different, which resulted in different compound stabilities.

Meanwhile, the negative Δ*H* values of naringin and naringin palmitate indicated that the formation of naringin-BSA/naringin palmitate-BSA coordination compound was an exothermic reaction. As shown by Table 3, the value of Δ*H* for naringin palmitate-BSA was lower than in naringin-BSA, while the value of Δ*G* was higher. This finding suggested that the forming compound of naringin-BSA was more stable than naringin palmitate-BSA.

### Binding Distances

**Figure 7:**
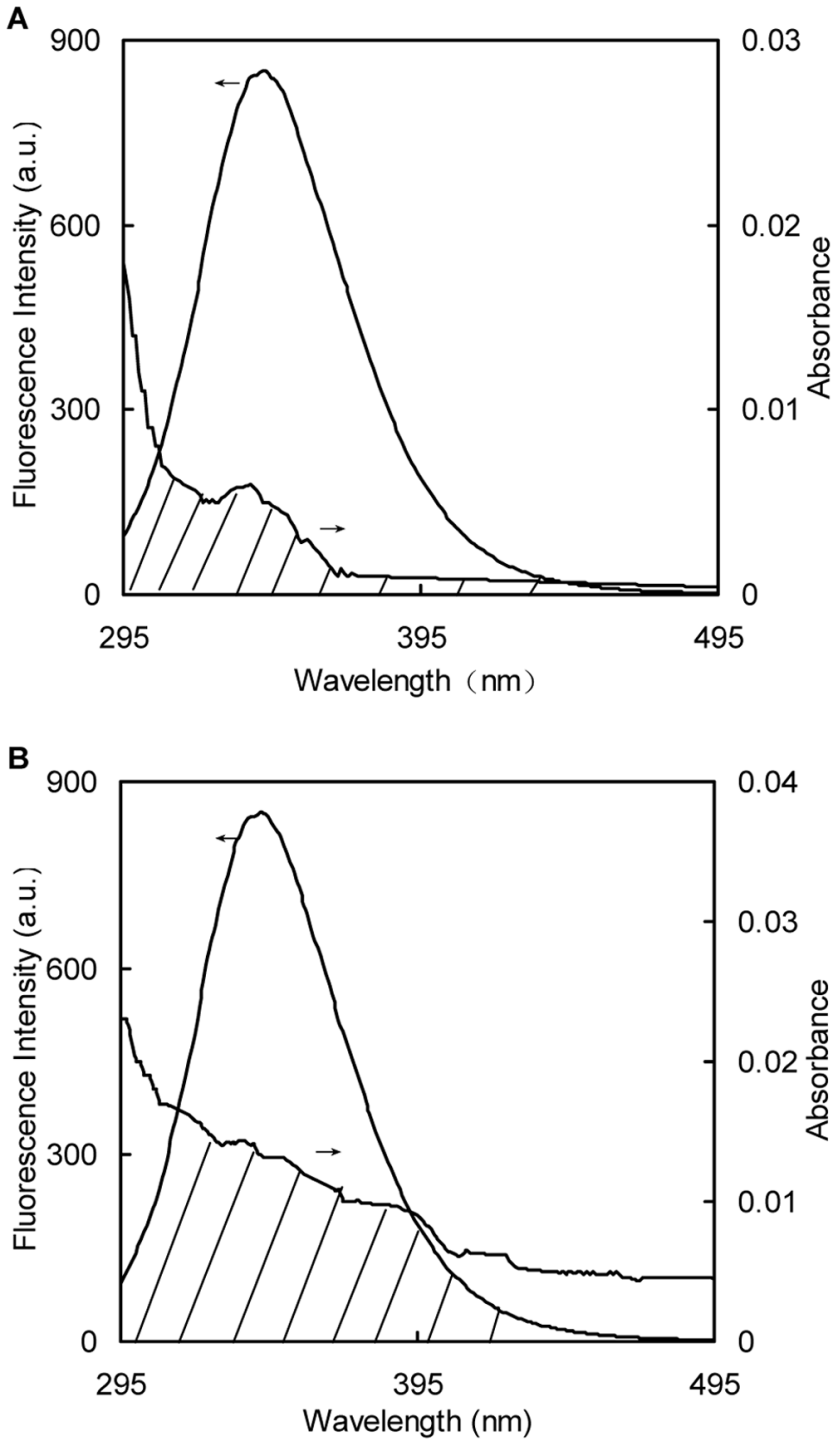
Spectral overlap of naringin and naringin palmitate absorption spectra with BSA fluorescence. (A) naringin and (B) naringin palmitate (*c*_BSA_ = *c*_naringin_ = *c*_naringin palmitate_ = 1.5×10^−6^ M).

To evaluate the distance between the ligands (naringin and naringin palmitate) and the tryptophan residues in BSA, the overlap of the UV/Vis absorption spectra of naringin and naringin palmitate with the fluorescence emission spectrums of BSA at 288 K were studied (Fig. 7). The spectral overlap integral between the fluorescence emission spectrum of the donor and the absorption spectrum of the acceptor (*J)*, the critical distance when the efficiency of energy transfer is 50%, the efficiency of energy transfer (*E)*, and the distances between the donor and acceptor (*r)* were calculated with Eq. 7, 6, 5 and 5, respectively (Table 4).

**Table 4:** Distance between BSA and naringin or naringin palmitate.

Sample	*J*/(cm^3^·L·mol^−1^)	*R*_c_/(nm)	*E*	*r*/(nm)
Naringin	3.397×10^−15^	2.13±0.018	0.038±0.008	3.64±0.071
Naringin palmitate	1.201×10^−14^	2.63±0.011	0.099±0.006	3.81±0.025

According to Föster’s non-radiative energy transfer theory, there are main influence factors that influence the efficiency of energy transfer, including the fluorescence production of the donor, the overlap between the fluorescence emission spectrum of the donor and the UV/Vis absorbance spectrum of the acceptor, and the distance between the donor and the acceptor, which is approached and lower than 8 nm [34]. The distances between naringin or naringin palmitate and BSA were 3.64 nm and 3.81 nm (Table 4), illustrating the energy transfer from BSA to naringin and naringin palmitate, as well as progress towards BSA the fluorescence quenching of BSA by naringin and naringin palmitate [54]. The donor-acceptor distance for naringin palmitate-BSA was larger than that for naringin-BSA, indicating the less stable binding in naringin palmitate-BSA.

### Analysis of Site Marker Competitive Experiments

**Figure 8:**
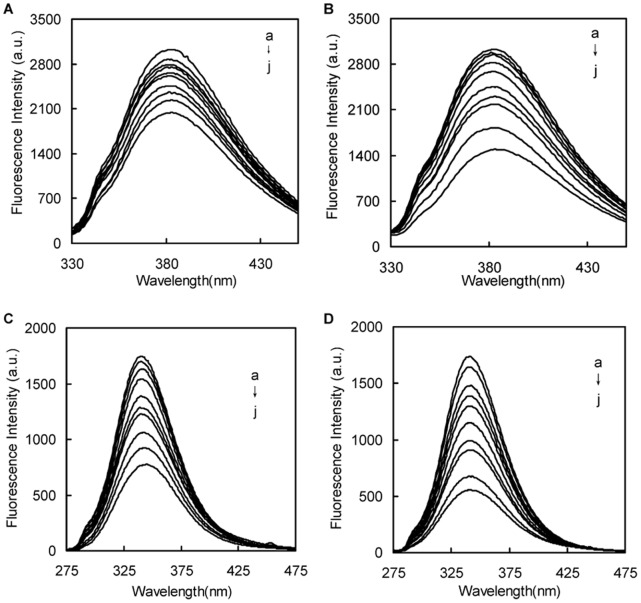
Displacement of warfarin and ibuprofen from BSA-warfarin and BSA-ibuprofen complex by naringin and naringin palmitate. (A) Displacement of warfarin from BSA-warfarin complex by naringin (B) Displacement of warfarin from BSA-warfarin complex by naringin palmitate (C) Displacement of ibuprofen from BSA-ibuprofen complex by naringin (D) Displacement of ibuprofen from BSA-ibuprofen complex by naringin palmitate (*c*_BSA_ = *c*_warfarin_ = 1.5×10^−6^ M, *c*_ibuprofen_ = 1.0×10^−6^ M, a→j: *c*_naringin_, *c*_naringin palmitate_ = 0, 1.5, 2.5, 5.0, 10, 15, 20, 30, 40, 50×10^−6^ M)).

It is well known that the principal binding sites in BSA are site I and site II, which are located in the hydrophobic cavities of subdomains IIA and IIIA, respectively [55]. Warfarin and ibuprofen are typical marker ligands for site I and site II respectively, while the molar ratio of ligand/protein is not larger than 1 [55]–[56]. In order to identify the binding sites of naringin-BSA and naringin palmitate-BSA, competitive binding studies were performed with warfarin and ibuprofen as site markers. The fluorescence intensity decreased with the increasing amounts of naringin and naringin palmitate in the case of warfarin-BSA and ibuprofen-BSA (Fig. 8), supporting the idea that the naringin and naringin palmitate were bound to the BSA [8]. The fluorescence quenching data for BSA in the presence of site markers were also analyzed with the Stern-Volmer equation (Eq. 1) [36]. The quenching constants *K_SV_* of BSA and the warfarin-BSA and ibuprofen-BSA systems were calculated from the slopes of three plots (Table 5).

**Table 5:** The quenching constants of BSA by naringin and naringin palmitate in competitive experiments.

Sample	Site maker	*10^4^×K*_sv_/(L·mol^−1^)	*R* a
Naringin	blank	3.762±0.022	0.980
	Warfarin	0.723±0.030	0.989
	ibuprofen	2.398±0.033	0.984
Naringin palmitate	blank	3.650±0.061	0.978
	Warfarin	1.408±0.041	0.992
	ibuprofen	4.201±0.045	0.987

a R is correlation coefficient.

For naringin, the *K_SV_* of the warfarin-BSA system was just 19.22±0.68% of free BSA, while the value of the ibuprofen-BSA system was almost 63.74±0.51% of free BSA (Table 5). There was significant competition between naringin and warfarin, while competition with ibuprofen was relatively low. These results suggested that naringin was preferentially bound to site I (subdomain IIA), where the warfarin was bound. For naringin palmitate, the *K_SV_* of the warfarin-BSA system was almost 38.58±0.46% of free BSA, while the *K_SV_* of ibuprofen-BSA system was larger than in the free BSA (Table 5). This finding implies that naringin palmitate was only bound to site I (subdomain IIA), where warfarin binds, which could be attributed to the large hydrophobic cavity within site I of BSA [55]. The *K_SV_* of the naringin palmitate-BSA-warfarin system was larger than in the naringin-BSA-warfarin system (Table 5), indicating that the competitive combining capacity of naringin palmitate is weaker than in naringin, which is in accordance with data indicating that there is 1.1 binding sites for naringin and 0.8 binding sites for naringin palmitate in BSA.

Hence, the above experimental results and analyses demonstrated that naringin was mainly located within site I (sub-domain IIA) and just partially overlapped site II (sub-domain IIIA) of BSA and that naringin palmitate was partially located within site I (sub-domain IIA) of the BSA.

### Conformation Investigation

**Figure 9:**
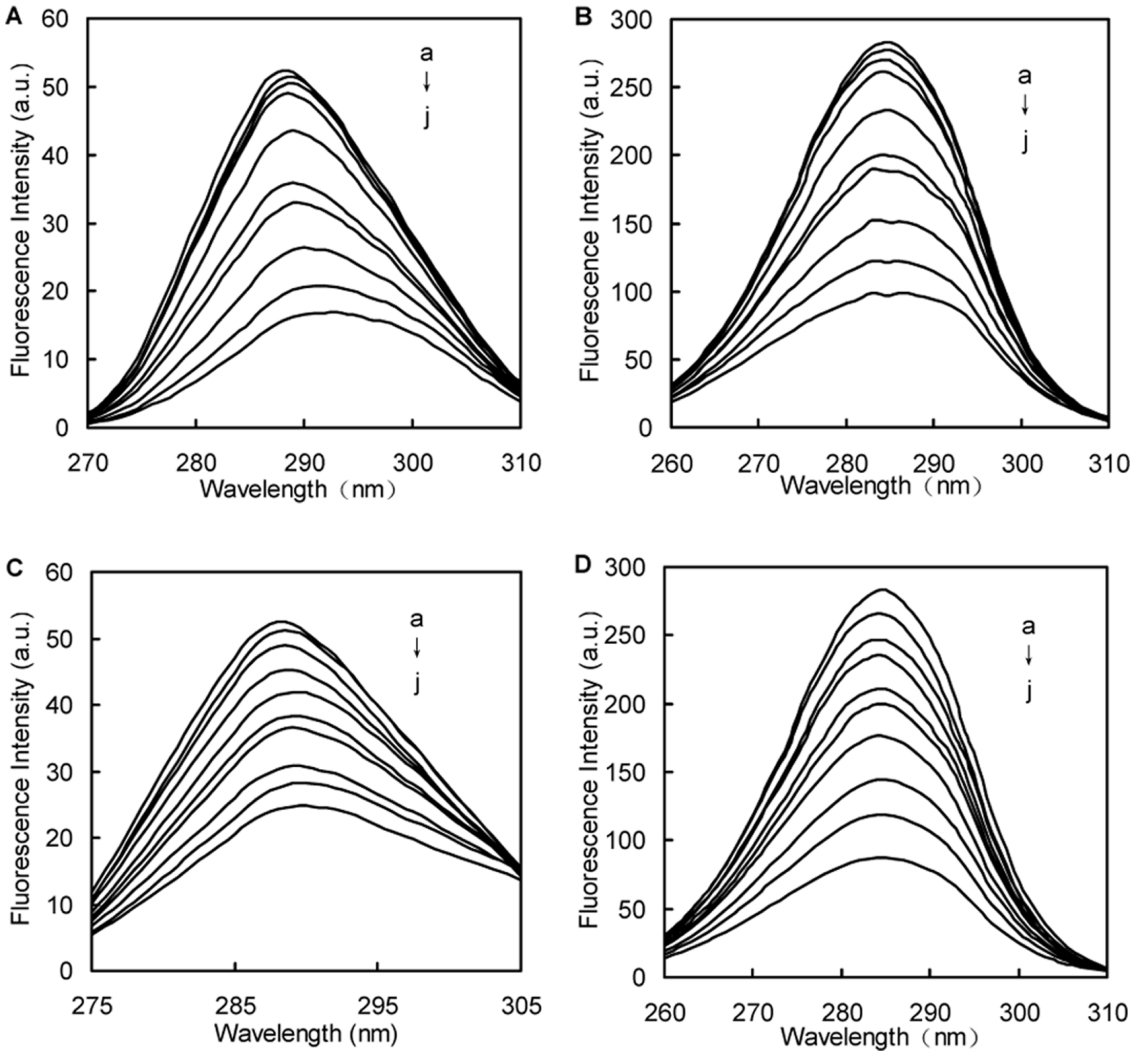
Synchronous fluorescence spectra for the interaction between naringin/naringin palmitate and BSA (Δ*λ* = 15 nm, 60 nm). (A) naringin (Δ*λ = *15 nm), (B) naringin (Δ*λ = *60 nm), (C) naringin palmitate (Δ*λ = *15 nm), (D) naringin palmitate (Δ*λ = *60 nm), (*c*_BSA_ = 1.5×10^−6^ M, a→j: *c*_naringin_, *c*_naringin palmitate_ = 0, 1.5, 2.5, 5.0, 10, 15, 20, 30, 40, 50×10^−6^ M)).

To explore the structural change of BSA from naringin palmitate and naringin, the synchronous fluorescence spectra of BSA at 288 K were measured with various amounts of naringin palmitate and naringin (Fig. 9). The synchronous fluorescence studies were conducted in order to provide information about the molecular environment in the vicinity of the chromosphere molecule [57]. The intrinsic fluorescence of many proteins is largely due to the tyrosine and tryptophan residues alone [17]. The characteristic information from tyrosine and tryptophan residues can be found by synchronous fluorescence when the *D*-value (Δ*λ*) between the excitation and emission wavelength were stabilized at 15 and 60 nm [58]. The influence of naringin and naringin palmitate on the structure of BSA is shown in Fig. 9 by synchronous fluorescence spectroscopy with Δ*λ* = 15 nm and Δ*λ* = 60 nm. A small red shift (from 288 to 290 nm) of tyrosine residues fluorescence (Fig. 9 C) and a small blue shift (from 285 nm to 284 nm) of tryptophan residues fluorescence (Fig. 9 D) appeared with the increasing amounts of naringin palmitate. It was also apparent that the red shift (from 288 to 293 nm) of tyrosine residues fluorescence (Fig. 9 A) and the small blue shift (from 285 nm to 283 nm) of tryptophan residues (Fig. 9 B) occurred with increasing amounts of naringin. The tyrosine residue shifts for naringin-BSA and naringin palmitate-BSA were more notable than in the tryptophan residues. The BSA conformation changes, which were accomplished by adding naringin and naringin palmitate, mainly resulted from increasing the polarity around the tyrosine residues and decreasing the hydrophobicity of the tyrosine residues [59].

**Figure 10:**
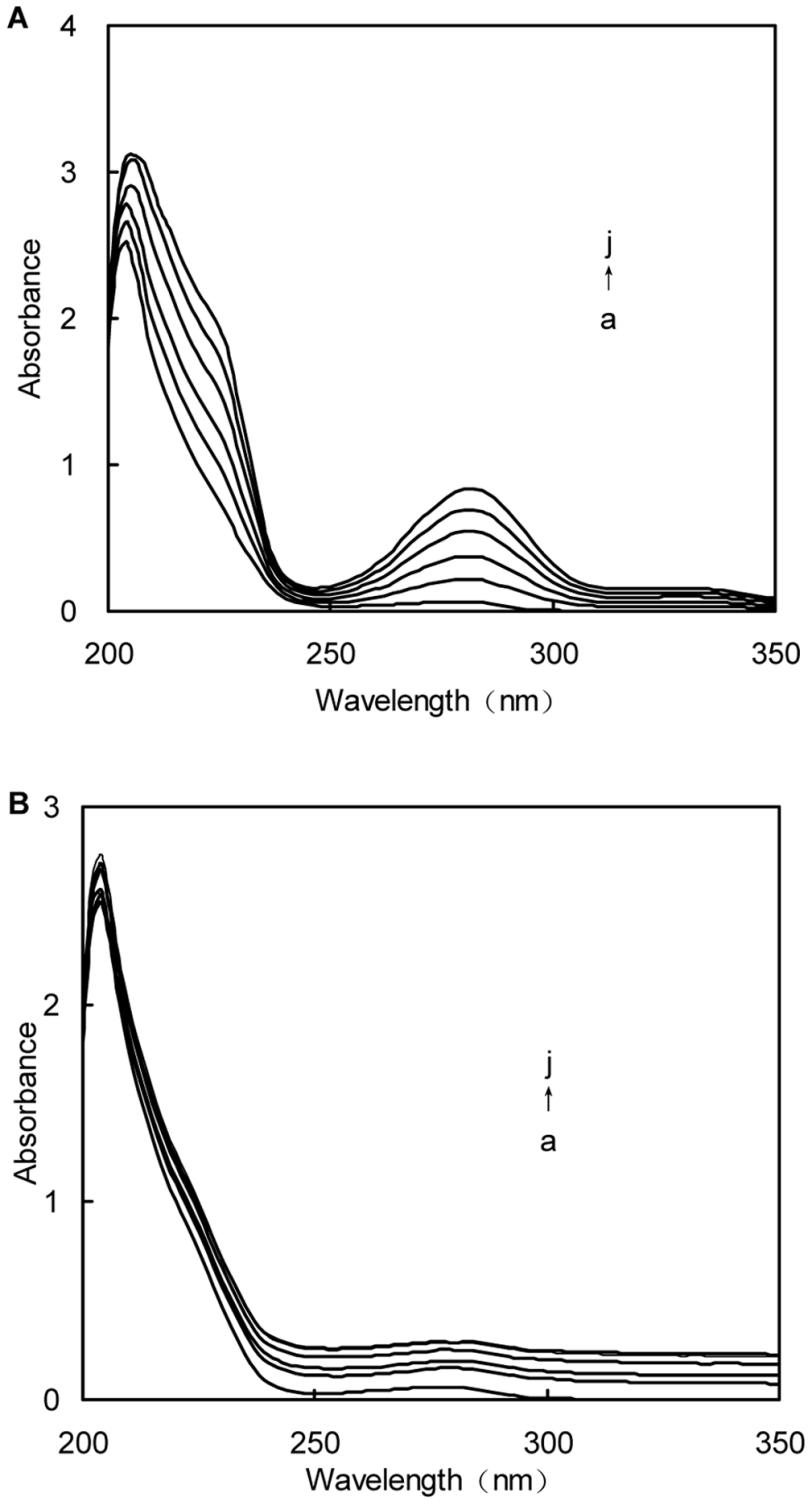
UV absorption spectra for the interaction between naringin/naringin palmitate and BSA. (A) naringin and (B) naringin palmitate (*c*_BSA_ = 1.5×10^−6^ M, a→j: *c*_naringin_ = 0, 1, 2, 3, 4, 5×10^−5^ M).

To reconfirm the structural change of BSA by adding various amounts of naringin and naringin palmitate, the UV/Vis absorbance spectra of BSA were measured, which indicated the change in hydrophobicity and the complex formation [60]–[61]. Fig. 10 shows that the UV/Vis absorbance spectra of BSA changed with different concentrations of naringin and naringin palmitate at 288 K. The fluorescence quenching of BSA was static quenching and the naringin-BSA and naringin palmitate-BSA complexes were then formed, which was confirmed by noting the change in the UV/Vis absorbance spectra [62]. The far-UV region of the spectra corresponds to secondary structures, and the absorption band of BSA (around 205 nm) represents the α-helix structure of BSA [19], [24], [63]. As seen in Fig. 10A, the UV absorption intensity of BSA around 205 nm increased regularly with the variation of naringin palmitate and naringin concentrations, indicating that the conformation of BSA was changed and complexes were formed between naringin/naringin palmitate and BSA [64]. The maximum peak position (λ_max_) of naringin palmitate-BSA did not experience a significant shift when the λ_max_ of naringin-BSA shifted slightly towards higher wavelength regions (from 278 nm to 282 nm). Valeur et al. pointed out that the change in λ_max_ indicated a change in polarity around the tryptophan residue, which would change the peptide strand of BSA molecules and the hydrophobicity [65]. Therefore, the results from Fig. 10 indicated that the addition of naringin palmitate did not change polarity around the tryptophan residue of BSA, or the change was so small it could not be detected. These results were consistent with the results of synchronous fluorescence spectra, suggesting that the local environment around the BSA tyrosine residues were changed with the addition of naringin palmitate when the local environment around BSA tryptophan and tyrosine residues had both been changed with the addition of naringin. These observations signified that the peptide strands of BSA molecules were more extended and the hydrophobicity was decreased with the addition of naringin palmitate [60]. These results were consistent with hesperidin-BSA binding results [18].

**Figure 11:**
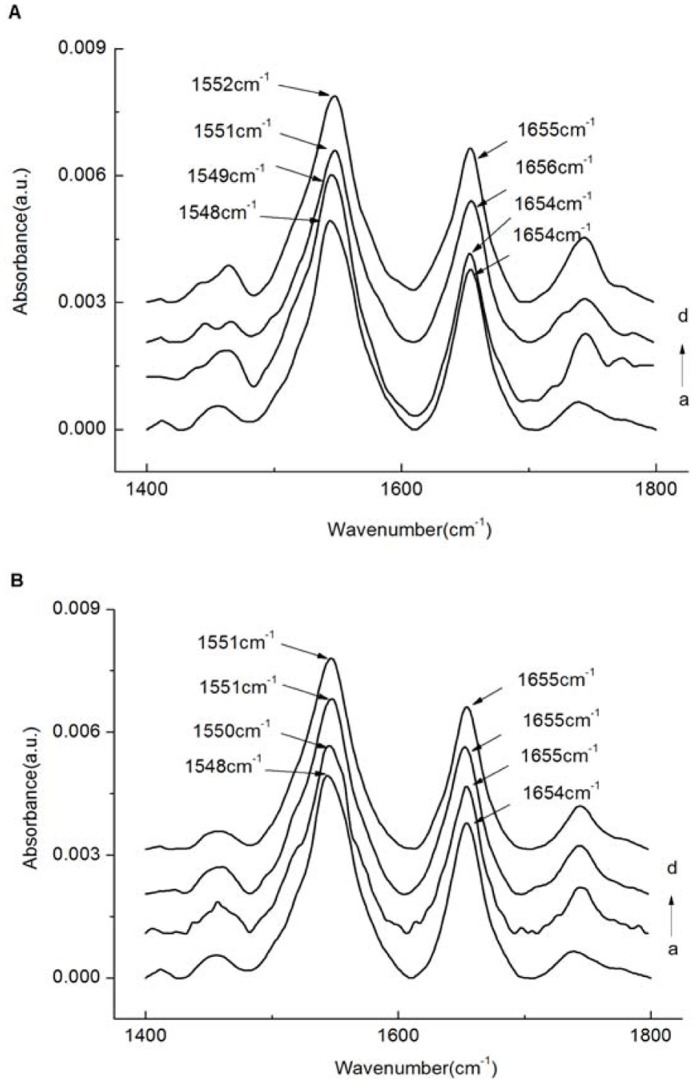
FT-IR spectra of BSA and BSA bound with naringin/naringin palmitate in the region 1800-1400 cm^−1^. (A) 0, 1.5, 3.0, 4.5×10^−6^ M (curves a→d) of naringin treated with BSA (1.50×10^−6^ M) and (B) 0, 1.5, 3.0, 4.5×10^−6^ M (curves a→d) of naringin palmitate treated with BSA (1.50×10^−6^ M).

FT-IR spectroscopy was used (Fig. 11) to obtain more information about the conformational changes of BSA after binding with naringin/naringin palmitate because the infrared spectra of the proteins exhibited amide bands (I–III) [54], which represented different peptide moiety vibrations [7]. The secondary structure of proteins was determined to occur at 1650–1660 cm^−1^ (amide I, principally due to C = O stretching) and 1548–1560 cm^−1^ (amide II, due to C–N stretching coupled with N–H bending) [66]. Generally, the 1650–1660 cm^−1^ range of the amide I band was attributed to the α-helix [67], which was used to predict the secondary structure of proteins [54]. In the present study, the characteristic amide I and amide II bands of free BSA were found at 1654 and 1548 cm^−1^, respectively. The peak position of amide I moved from 1654 cm^−1^ to 1656 cm^−1^, and for amide II, it moved from 1548 cm^−1^ to 1552 cm^−1^, upon binding with naringin (Fig. 11A). The peak position of amide I moved from 1654 cm^−1^ to 1655 cm^−1^, and for amide II, it moved from 1548 cm^−1^ to 1551 cm^−1^, upon complexation with naringin palmitate (Fig. 11B). These results showed the BSA secondary structure effect in relation to its complexation with naringin and naringin palmitate [7], [18], resulting in the perturbations of amide I and amide II vibrational frequencies [68]–[69]. Additionally, the conformational changes of naringin palmitate-BSA were less than in naringin-BSA, suggesting the greater influence of naringin on the structure of BSA.

**Figure 12:**
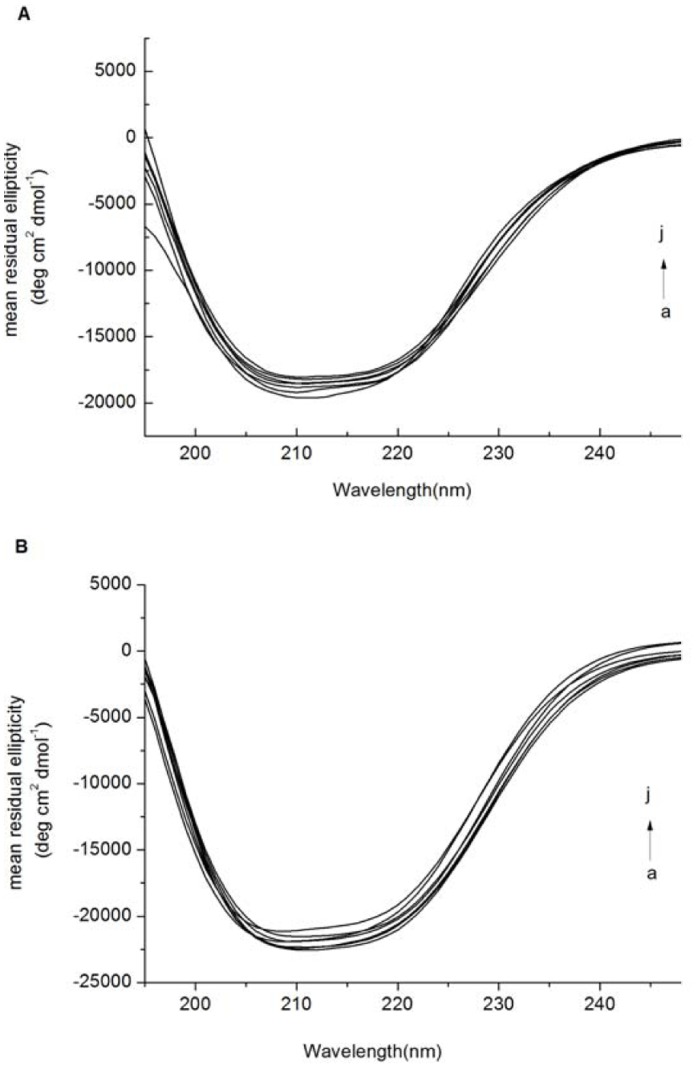
CD spectra of free BSA and complexed with naringin and naringin palmitate. (A) 0, 3.0, 6.0, 9.0, 12.0, 15.0, 2.25×10^−6^ M (curves a→j) of naringin treated with BSA (1.50×10^−6^ M) and (B) 0, 3.0, 6.0, 9.0, 12.0, 15.0, 22.5×10^−6^ M (curves a→j) of naringin palmitate treated with BSA (1.50×10^−6^ M).

To clarify the specific changes in the BSA α-helix structure after binding with naringin/naringin palmitate, the far-UV CD spectra of BSA were studied in the presence of different amounts of naringin/naringin palmitate [39], [70] (Fig. 12). Two negative bands at 208 nm and 222 nm in the ultraviolet region of the BSA CD spectrum were characteristic of the α-helical structure of a protein [39]. An obvious decrease in band intensity appeared with the addition of naringin and naringin palmitate, indicating the destabilization of BSA helical structures with an increase of naringin and naringin palmitate, suggesting considerable changes in the protein secondary structure (Fig. 12) [39], [70].

The MRE at 208 nm was regarded as an indicator of α-helix structure in proteins [9], [17]. The α-helical content of free BSA was calculated from the mean residue ellipticity values at 208 nm, which was 66.1±0.2%, as similarly reported once before [17]. The α-helical content of naringin-BSA decreased to 63.1±0.1, 62.9±0.1, 62.7±0.2, 61.3±0.4, 59.9±0.3 and 56.1±0.5% with increasing concentrations (1∶2, 1∶4, 1∶6, 1∶8, 1∶10 and 1∶15), while the α-helical content of naringin palmitate-BSA decreased to 64.9±0.2, 64.5±0.1, 62.4±0.3, 61.7±0.2, 60.8±0.5 and 60.1±0.2% accordingly (Fig. 12), which was in agreement with the values reported by other investigators [17], [44]. It can be concluded that the conformation of BSA changed with the reduction of Θ-helical content in the presence of naringin and naringin palmitate. There was also a more obvious naringin influence on the conformation of BSA than in naringin palmitate, which was consistent with the FT-IR results, indicating the higher binding capacity of naringin to BSA than naringin palmitate.

### Molecular Modelling and Docking

**Figure 13:**
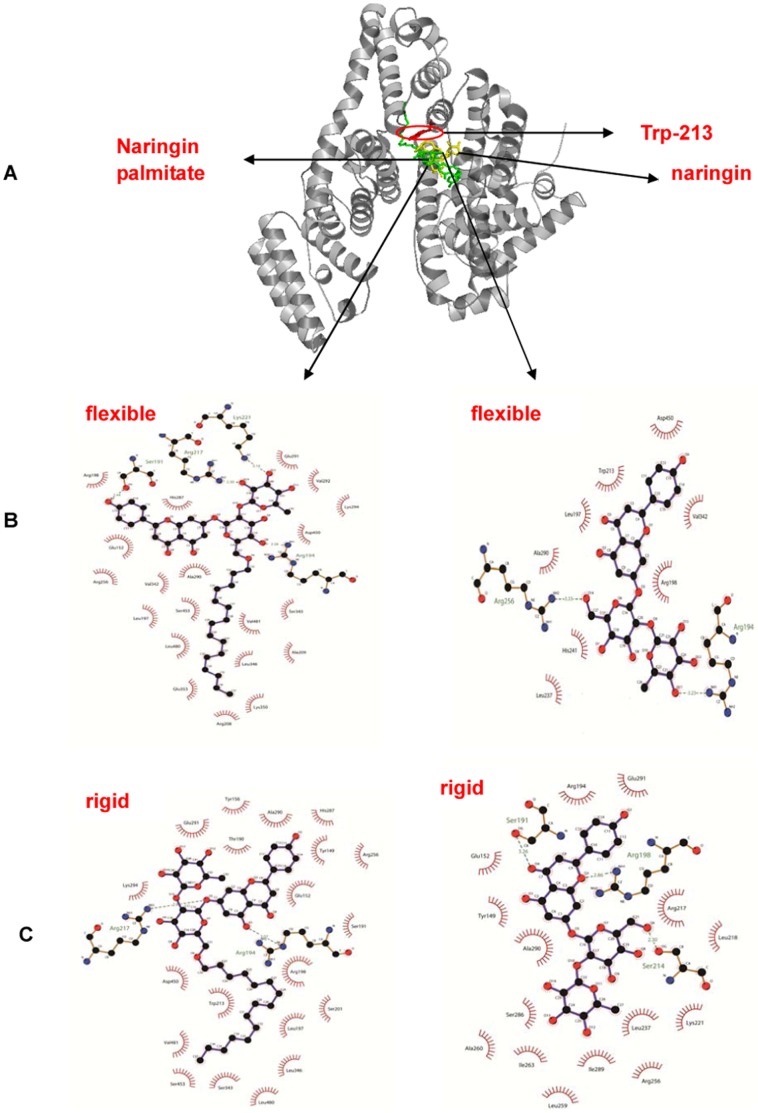
Molecular contacts between the amino acid residues of the site of BSA with naringin and naringin palmitate. Obtained from rigid and flexible docking by AutoDock 4.2 are shown in the form of LigPlot: (A) Schematic representation of BSA molecule (Trp-213 is in red, naringin is in yellow and naringin palmitate is in green), (B) flexible docking of naringin and naringin palmitate. (C) rigid docking of naringin and naringin palmitate.

In order to investigate the interactions between substrates and BSA, both rigid and flexible docking studies were performed by AutoDock 4.2 [24]. The most likely substrate binding modes were obtained by extracting the lowest energy conformation from the largest cluster (Fig. 13). The flexible docking results clearly suggested that naringin was absolutely bound to Sudlow’s site I (subdomain IIA) where Trp-213 residue located, while naringin palmitate was partially bound to that site (Fig. 13A). These conformations were consistent with the results of prior site marker experiments and the number of binding sites for naringin (1.1) and naringin palmitate (0.7–0.8). Within the flexible docking binding sites of naringin, there were possible hydrogen bond interactions between the 18-OH of naringin and Arg-256 (3.33 Å) and between the 23-OH of naringin and Arg-194 (3.23 Å) (Fig. 13B). In addition, residues Trp-213, Leu-197, Leu-237 and Val-342 could provid extra hydrophobic force to stabilize the naringin-BSA complex (Fig. 13B). Meanwhile, the existence of many charged and polar residues (Arg-194, Arg-198, Arg-256, His-241 and Leu-237) could play a subordinate role in stabilizing the naringin molecule via electrostatic interactions (Fig. 13B). These results suggested that naringin was bound to BSA with electrostatic, hydrophobic and hydrogen bond interactions, which was in agreement with the binding model above. A similar observation for naringin-BSA was previously reported [8].

On the other hand, the most likely binding conformation for naringin palmitate is significantly different from naringin in terms of flexible docking binding (Fig. 13B). Naringin palmitate was located in the mouth of subdomain IIA and was slightly apart from residue Trp-213 when compared with naringin, and the long alkyl chain was located on the surface of subdomain IIA. This finding is supported by the different binding distance and sites. Four hydrogen bond interactions existed between the 4-OH and residue Ser-191 (2.44 Å), the 24-OH and residue Lys-221 (3.14 Å), the 25-OH and residue Arg-217 (2.30 Å), and the 15-OH and residue Arg-194 (2.58 Å). Hydrophobic residues such as Leu-197, Leu-346, Leu-480, and Val-342 surround naringin palmitate, which could enhance the binding affinity of naringin palmitate with BSA. However, the interaction forces for binding naringin palmitate and BSA were van der Waals force and hydrogen bonding formation, which were derived from the thermodynamics of this interaction. Therefore, hydrophobic interactions were not the main interaction forces for binding the naringin palmitate-BSA compound. The molecular docking and thermodynamics suggested that naringin palmitate was mainly bound to BSA with hydrogen bond interactions. These hydrogen interactions would decrease hydrophilicity and increase hydrophobicity [71]–[72], resulting in the stabilization of the naringin palmitate-BSA complex. Although naringin palmitate was only partially bound to the BSA, any perturbation may induce changes in subdomain IIA as well [73], so fluorescence quenching occurred.

**Figure 14:**
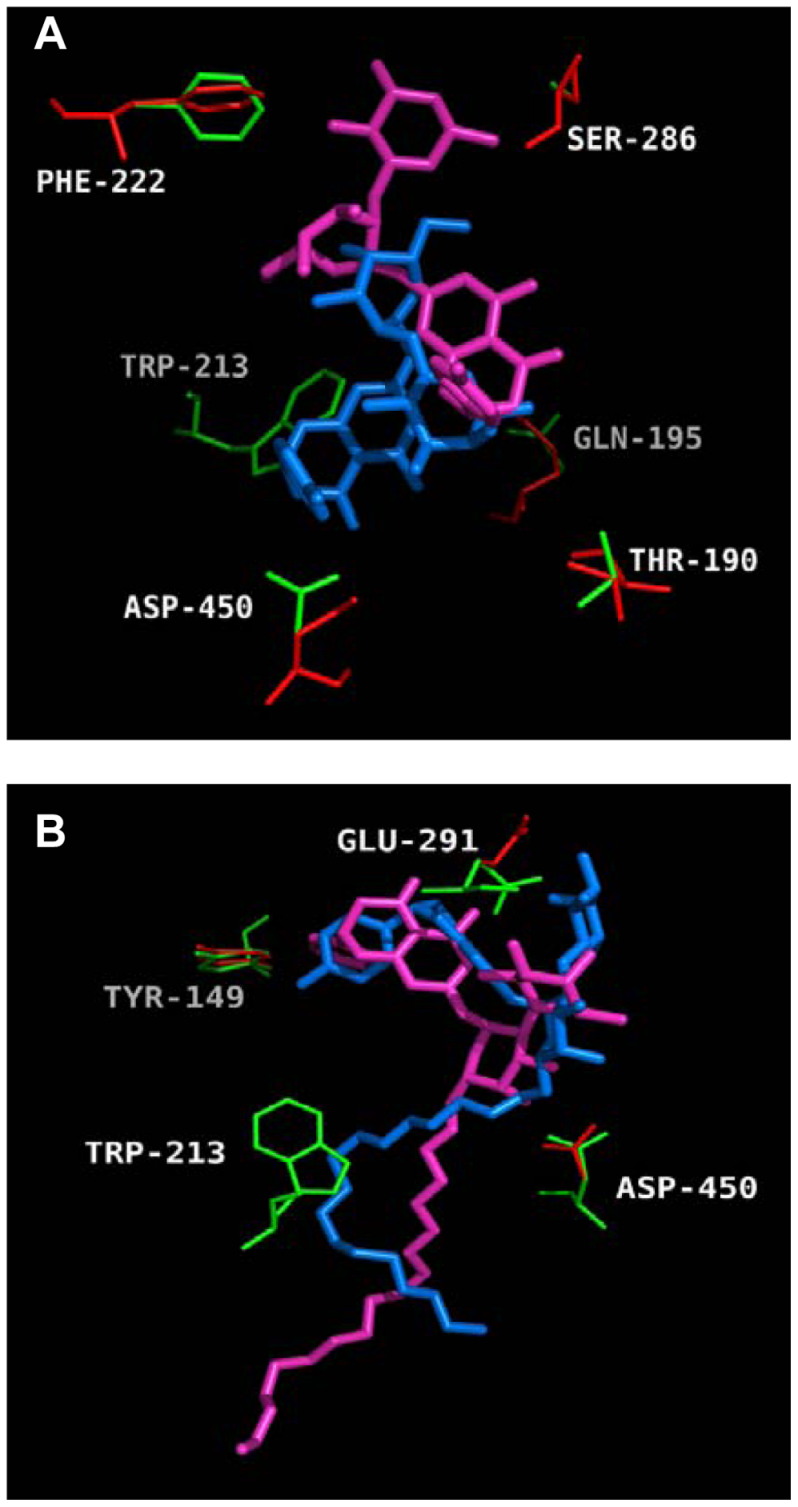
Molecular docking of BSA with naringin and naringin palmitate. Residues in flexible (red) and rigid (green) modes are shown: (A) naringin, (B) naringin palmitate. In flexible docking the ligands are in magenta and in rigid docking they are in blue color.

The conformations that were obtained through rigid and flexible docking studies were compared in Figs. 14. The overall conformations of ligands were different to a certain extent. This difference was a consequence of the high flexibility of the ligands, i.e. naringin and naringin palmitate. Although the distances between Trp-213 and naringin were similar in both rigid and flexible docking studies, the hydrophobic force contribution of Trp-213 was only identified in the flexible mode. On the other hand, the conformation of the naringin palmitate-BSA complex, which was obtained by flexible docking, also seems reasonable because the distance between Trp-213 and naringin palmitate (5.3 Å) was consistent with the results of the notable shift in fluorescence studies, where the distance identified in rigid mode was 2.6 Å (not shown). Therefore, the side-chain flexibility of specific residues may help to identify optimal theoretical conformations.

The interaction between naringin palmitate and BSA has been studied in great depth using fluorescence quenching, UV absorption spectroscopy, FT-IR, CD spectroscopy, site marker experiments and molecular modelling methods. This work demonstrates that naringin palmitate can bind to BSA and that the addition of naringin palmitate to the buffer solution of protein changes the conformation of BSA, which is substantiated by the CD and FT-IR spectra data. The quenching mechanism of BSA fluorescence by naringin palmitate is static, which is similar to naringin. The temperature affects the stability of the naringin palmitate-BSA complex, and the naringin palmitate and BSA compound is more sensitive to high temperature than naringin-BSA. Displacement experiments and molecular modelling studies suggest that naringin palmitate only partially interacts with site I of the BSA, and the main interaction between them is van der Waals force and hydrogen bonding formation. The distance between naringin palmitate and the BSA binding site is larger than that of naringin, so the naringin palmitate-BSA is less stable than naringin-BSA. In summary, naringin palmitate can bind to BSA and be released more quickly from BSA than naringin, which quickly reaches the maximum concentration. Naringin palmitate can also be transported by proteins and removed easily from the body, and when naringin palmitate is used in a mammal body, it will be effective in improving short-term clinical treatments.
